# Boosting the effects of hyperthermia-based anticancer treatments by HSP90 inhibition

**DOI:** 10.18632/oncotarget.22142

**Published:** 2017-10-27

**Authors:** Lianne E.M. Vriend, Nathalie van den Tempel, Arlene L. Oei, Mike L’Acosta, Frederique J. Pieterson, Nicolaas A.P. Franken, Roland Kanaar, Przemek M. Krawczyk

**Affiliations:** ^1^ Department of Medical Biology, Meibergdreef 15, 1105 AZ Amsterdam, The Netherlands; ^2^ Department of Radiation Oncology, Laboratory of Experimental Oncology and Radiobiology (LEXOR), Meibergdreef 15, 1105 AZ Amsterdam, The Netherlands; ^3^ Cancer Center Amsterdam, Meibergdreef 15, 1105 AZ Amsterdam, The Netherlands; ^4^ Department of Molecular Genetics, Cancer Genomics Center Netherlands, Department of Radiation Oncology, Erasmus Medical Center, ‘s-Gravendijkwal 230, 3015 CE Rotterdam, The Netherlands

**Keywords:** hyperthermia, HSP90 inhibition, DNA repair, thermotolerance, ganetespib

## Abstract

Hyperthermia – application of supra-physiological temperatures to cells, tissues or organs – is a pleiotropic treatment that affects most aspects of cellular metabolism, but its effects on DNA are of special interest in the context of cancer research and treatment. Hyperthermia inhibits repair of various DNA lesions, including double-strand breaks (DSBs), making it a powerful radio- and chemosensitizer, with proven clinical efficacy in therapy of various types of cancer, including tumors of head and neck, bladder, breast and cervix. Among the challenges for hyperthermia-based therapies are the transient character of its effects, the technical difficulties in maintaining uniformly elevated tumor temperature and the acquisition of thermotolerance. Approaches to reduce or eliminate these challenges could simplify the application of hyperthermia, boost its efficacy and improve treatment outcomes. Here we show that a single, short treatment with a relatively low dose of HSP90 inhibitor Ganetespib potentiates cytotoxic as well as radio- and chemosensitizing effects of hyperthermia and reduces thermotolerance in cervix cancer cell lines. Ganetespib alone, applied at this low dose, has virtually no effect on survival of non-heated cells. Our results thus suggest that HSP90 inhibition can be a safe, simple and efficient approach to improving hyperthermia treatment efficacy and reducing thermotolerance, paving the way for *in vivo* studies.

## INTRODUCTION

DNA-damaging agents, such as ionizing radiation, topoisomerase inhibitors, DNA intercalators or cross-linkers, are among the most effective anticancer modalities exploited in diverse clinically relevant therapies. However, the intricate DNA repair mechanisms that evolved to maintain the integrity of genetic information of healthy cells, protect DNA of cancer cells, effectively increasing their resistance to therapy [1].

DNA double-strand breaks (DSBs) are arguably the most dangerous DNA lesions induced by anticancer treatments. In mammalian cells, DSB repair is executed by two major pathways, called non-homologous end joining (NHEJ) and homologous recombination (HR). NHEJ is a robust and conceptually simple mechanism, active throughout the cell cycle. The pathway involves a direct rejoining of the broken DNA ends, often at the cost of inducing nucleotide deletions or insertions [2]. In contrast to NHEJ, HR is a more complex and precise mechanism – relying on BRCA1, BRCA2 and RAD51, among other proteins – that can utilize an intact DNA fragment as a repair template [3]. The activity of HR is tightly coupled to cell cycle progression and limited to the S and G_2_ phases of the cell cycle. Given the involvement of DSB repair in the resistance to DNA-damaging agents, its inactivation in cancer cells could increase their sensitivity to therapy. Despite considerable efforts, however, safe, potent, selective and bioavailable inhibitors of DSB repair have yet to emerge.

Hyperthermia – elevation of the tumor temperature above physiological levels, usually to 41-42.5°C – is a clinically applied anticancer therapy that affects multiple aspects of cellular metabolism, including DNA repair [4]. Hyperthermia is an excellent radiosensitizer and chemosensitizer, as demonstrated by *in vitro* and *in vivo* studies, as well as by randomized clinical trials [4–6]. One important feature of hyperthermia is that its application can generally be limited to the tumor volume, sparing the non-transformed surrounding tissues. Notably, hyperthermia efficiently inhibits HR, likely by inducing degradation of its essential protein BRCA2 [7, 8], as well as NHEJ, possibly in part by affecting DNA-PKcs or LIG4 [9, 10]. This may explain how hyperthermia sensitizes cells to agents such as ionizing radiation or cisplatin, because DNA lesions induced by these agents require HR and NHEJ for repair.

The radiosensitizing and chemosensitizing effects of hyperthermia are desirable in anticancer therapies, but they are counteracted by chaperone proteins that protect cells from the effects of various forms of stress, including heat. Heat-shock proteins (HSPs) are a subgroup of chaperone proteins that strongly respond to increased temperatures to regulate various genes and metabolic pathways as well as to physically protect their client proteins from heat-induced unfolding, inactivation and degradation [11]. One member of this group, HSP90, is of special interest in the context of cancer treatment and hyperthermia. HSP90 is an evolutionarily conserved chaperone, crucial in mammalian proteostasis, with affinity for a vast number of client proteins [12]. Inhibition of this chaperone affects the stability of some essential DNA repair factors, including BRCA1, BRCA2, RAD51, CHK1 and DNA-PKcs [13].

Recently, we reported that inhibition of HSP90 by 17-DMAG, the derivative of the antibiotic geldanamycin, can enhance the effects of hyperthermia on DSB repair, likely, at least in part, by stimulating hyperthermia-induced degradation of BRCA2 [7]. 17-DMAG also potentiates hyperthermic sensitization of cancer cells to PARP1 inhibition *in vitro* and *in vivo*. Importantly, the drug showed only limited cytotoxicity as a single agent, suggesting that HSP90 inhibition could be a safe and effective approach to potentiate effects of hyperthermia. In the current study, we focus on Ganetespib, a new-generation, more specific and well-tolerated HSP90 inhibitor that has been extensively studied *in vitro,* in animal models and in multiple clinical trials [14]. Since hyperthermia is routinely applied to a subset of cervical cancer patients, we use two cervical cancer cell lines, SiHa and HeLa to show that Ganetespib enhances the induction of DNA damage and cell killing by hyperthermia. Moreover, we demonstrate that Ganetespib potentiates hyperthermia-induced sensitization of cervix cancer cells to a number of DSB-inducing agents and reduces hyperthermia-induced thermotolerance, suggesting that HSP90 inhibition could be a safe, simple and effective strategy to improve the outcomes of clinical treatments involving hyperthermia.

## RESULTS

### HSP90 inhibitor Ganetespib potentiates the inhibitory effects of hyperthermia on HR

**Figure 1:**
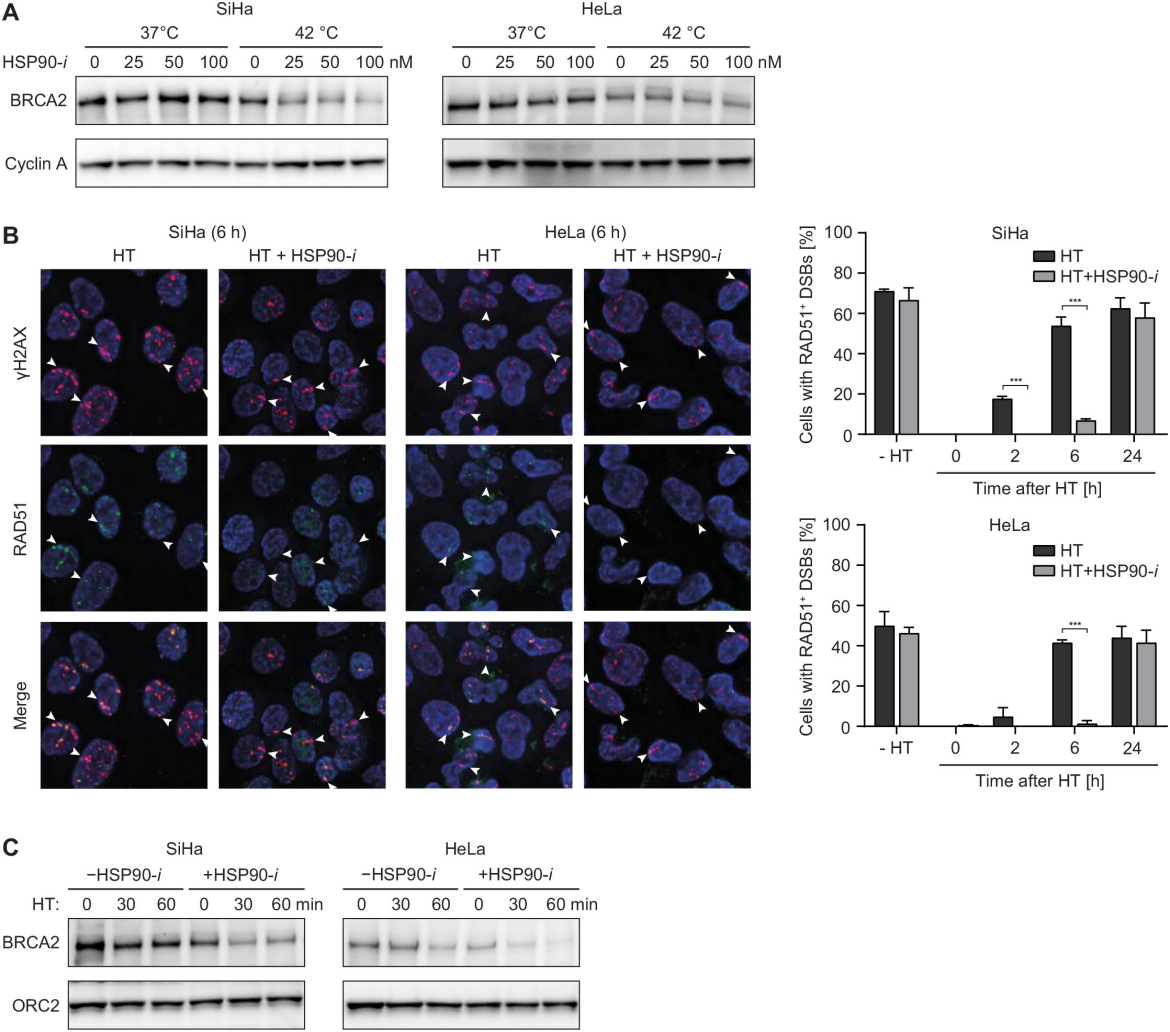
HSP90 inhibitor Ganetespib potentiates the effects of hyperthermia (HT) on HR **(A)** SiHa cells were incubated with the indicated concentrations of Ganetespib (HSP90-*i*) for 30 min at 37°C, then for an additional hour at 37 or 42°C. Next, cells were lysed and lysates were analyzed by western blotting, using antibodies against BRCA2 and Cyclin A (loading control). **(B)** Cells were treated as in (A), except Ganetespib was used at a concentration of 30 nM. At the indicated time after treatment, cells were irradiated with α-particles, fixed 30 min later and stained using antibodies against γH2AX (red) and RAD51 (green). The pictures are representative for cells irradiated 6 h after the treatment. The graphs show average percentage of cells containing tracks of γH2AX foci that were also positive for RAD51. **(C)** Cells were treated and analyzed as in (A), except the duration of incubation at elevated temperature was 0, 30 or 60 min. Equal sample loading was confirmed by probing for ORC2.

To investigate whether Ganetespib promotes the inhibitory effects of hyperthermia on DSB repair, we first analyzed hyperthermia-induced changes in the levels of BRCA2 protein. As expected, we found that treatment for 60 min at 42°C reduced the levels of BRCA2 (Figure 1A). Importantly, addition of Ganetespib further enhanced BRCA2 degradation in a dose-dependent manner. We found that a 1.5 h treatment with Ganetespib alone (up to 100 nM) had only modest effects on clonogenic cell survival, but this was enhanced after hyperthermia, at least at Ganetespib concentrations exceeding 3 nM (Supplementary Figure 1). We therefore decided to use the 30 nM concentration of Ganetespib in the subsequent experiments. One of the hallmarks of hyperthermia-induced HR deficiency is a disturbed accumulation of RAD51 at sites of DSBs [7, 8]. Indeed, we found that hyperthermia temporarily abolished recruitment of RAD51 to α-particle-induced DSBs. This effect was enhanced by Ganetespib, as RAD51 accumulation was impaired for considerably longer periods of time, in both SiHa and HeLa cells (Figure 1B). Treatment with Ganetespib alone did not affect RAD51 accumulation.

One of the most challenging aspects in successful clinical application of hyperthermia is the maintenance of the elevated tumor temperature for a sufficiently long period of time. Therefore, any approach to reduce the time required for efficient radio- or chemosensitization could greatly facilitate and boost hyperthermia treatments. To examine whether Ganetespib can shorten hyperthermia time required for efficient degradation of BRCA2, we heated HeLa and SiHa cells for 30 or 60 min, in the presence or absence of Ganetespib, and analyzed BRCA2 levels. Results show that a 30 min hyperthermia + Ganetespib combination treatment is at least as effective in reducing BRCA2 levels as a 60 min hyperthermia treatment without the inhibitor (Figure 1C). In summary, these results confirm that Ganetespib can potentiate the inhibitory effects of hyperthermia on HR in cervical cancer cells *in vitro*.

### Inhibition of HSP90 enhances induction of DNA damage by hyperthermia

**Figure 2:**
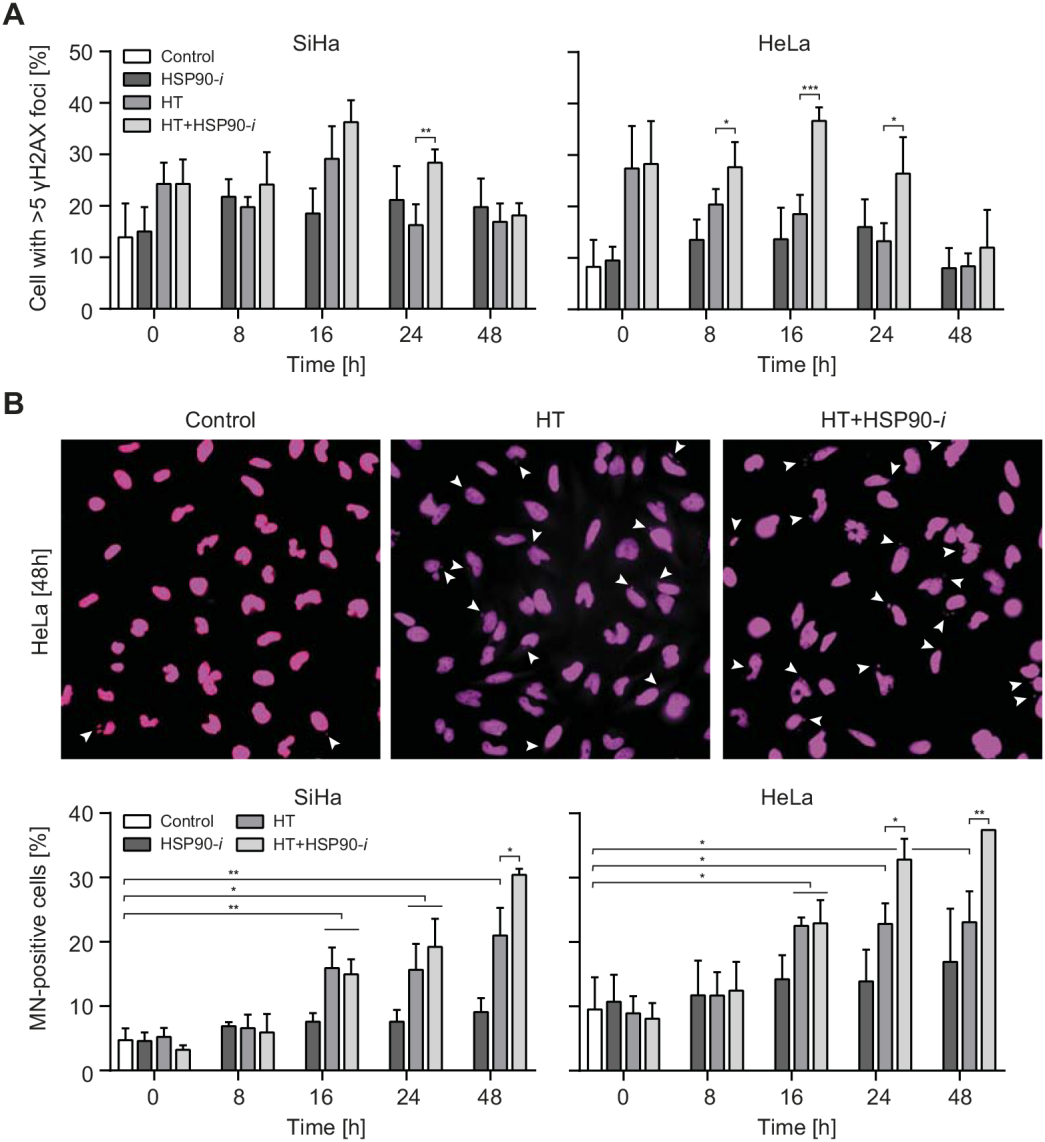
Inhibition of HSP90 enhances induction of DNA damage by hyperthermia (HT) Cells were incubated for 30 min with 30 nM Ganetespib (HSP90-*i*) at 37°C, then for 60 min at 37 or 42°C (HT). Medium was refreshed and cells were incubated at 37°C for the indicated time period, fixed and stained for γH2AX **(A)** and DNA **(B)**. (A) Average percentages of cells with more than 5 γH2AX foci after the indicated treatments. (B) The pictures (top panel) show micronuclei (MN)-containing HeLa cells at 48 h after treatments. The graphs (bottom panel) show the average frequencies of MN-containing (MN^+^) cells.

Hyperthermia has been suggested to induce DSBs in at least two ways [15]. First, it has been speculated that the elevated temperature induces damage directly, as heating leads to the focal accumulation of some repair factors, which are considered to mark sites of ongoing DSB repair [16, 17]. Second, hyperthermia has been suggested to stabilize the topoisomerase 1 (TOP1)-DNA cleavage complexes, which may lead to DSB formation in the next S-phase, when replication forks collide with single-stranded breaks (SSBs) induced by removal of the TOP1 [18]. To examine whether HSP90 inhibition can potentiate these effects as well, we first analyzed the induction of γH2AX foci at various time points (up to 48 h after hyperthermia treatment). We observed, in both cell lines, that hyperthermia led to increased frequencies of foci-containing cells immediately after treatment, and that these frequencies diminished at later time points (Figure 2A). After treatment with Ganetespib alone, the frequencies of γH2AX foci-positive cells were not affected immediately, but they did moderately increase at later time points (2-24 h), only to return to the baseline at 48 h after treatment. Combination of hyperthermia with Ganetespib led to a further increase of foci-positive cell frequencies, especially at later time points (at 16 and 24 h after treatment). γH2AX is an indirect marker of DSB formation and, especially after hyperthermia, it may mark sites of other lesions [19]. Therefore, we subsequently measured the induction of micronuclei (MN), which are a direct consequence of unrepaired DSBs [20] (Figure 2B). Our results show that hyperthermia alone increased MN^+^ cell frequency at >8 h after treatment, strongly suggesting induction of DSBs. In contrast, treatment with Ganetespib alone did not significantly affect MN^+^ cell numbers, whereas combinational treatment (hyperthermia + Ganetespib) led to an increased MN induction at later time points (at 24 and 48 h), as compared to hyperthermia alone (Figure 2B). The frequency of MN^+^ cells after the combinational treatment at 48 h was 3.5-fold (HeLa cells) and 6-fold higher (SiHa cells) than in untreated control cells. Combined, these results strongly suggest that hyperthermia does induce DSBs, probably indirectly, and that this effect is potentiated by HSP90 inhibition.

### Inhibition of HSP90 enhances radiosensitizing and chemosensitizing effects of hyperthermia

**Figure 3:**
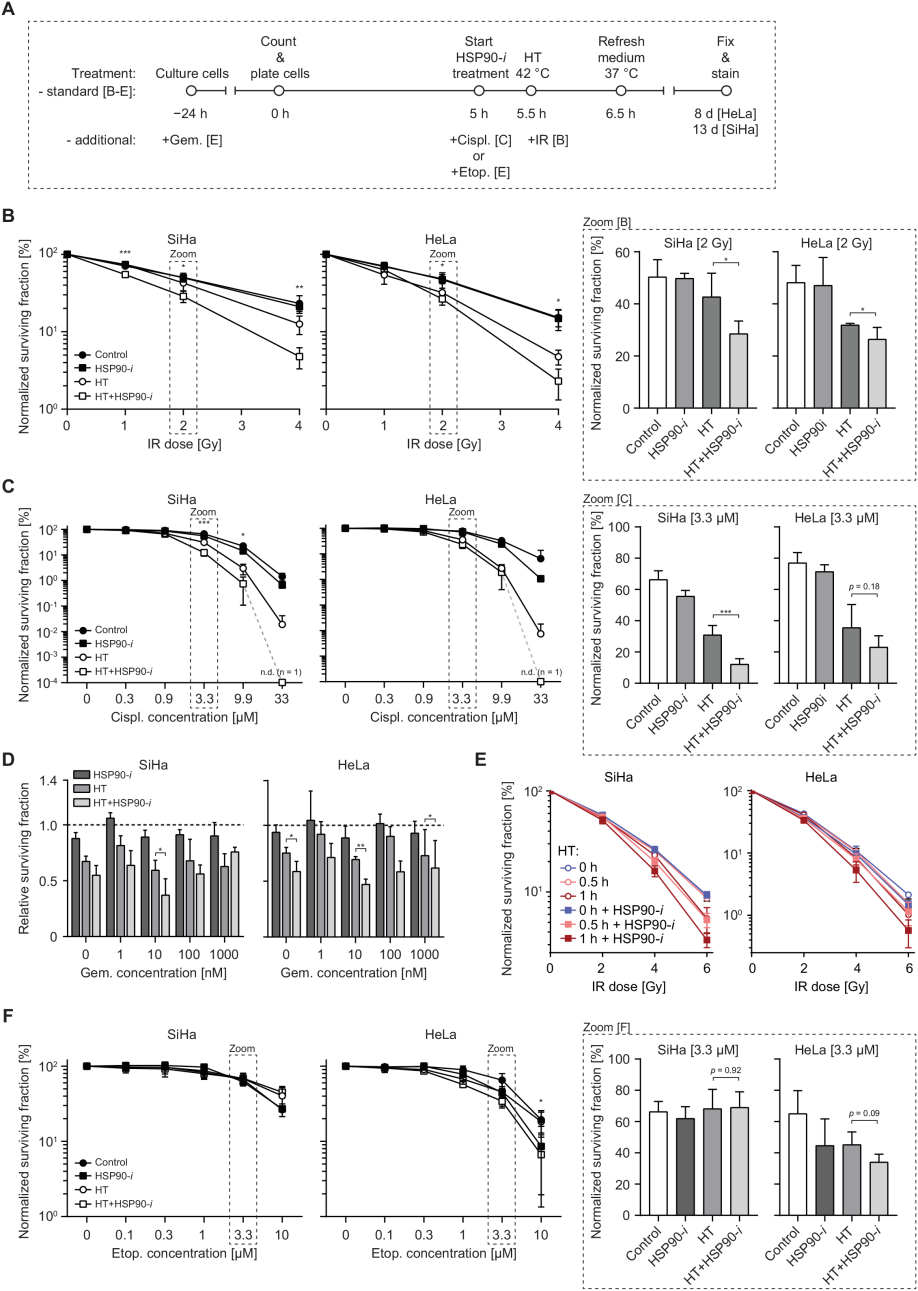
Inhibition of HSP90 enhances radiosensitizing and chemosensitizing effects of hyperthermia (HT) **(A)** Schematic overview of the treatment schedule for the clonogenic assay, results of which are presented in B-D and F. **(B)** Normalized clonogenic survival after increasing doses of IR alone or combined with HT and/or Ganetespib (HSP90-*i*). Bar graphs (right-hand panels outlined by the dashed lines) show the enlargement of the data points from the 2 Gy dose. **(C)** Normalized clonogenic survival after increasing concentration of cisplatin (Cispl.) alone or combined with HT and/or HSP90-*i*. At the highest concentration (33 μM) no clones were detectable (n.d.). Bar graphs (outlined by dashed lines) show the enlargement of the data points from the 3.3 μM concentration. **(D)** Relative clonogenic survival after increasing concentration of gemcitabine (Gem.) alone or in combination with HT and/or HSP90-*i*. The dotted line indicates the cell survival after treatment with gemcitabine alone. **(E)** Normalized clonogenic survival of cells incubated at 42°C for the indicated period of time, in the presence or absence of HSP90-*i* and exposed to the indicated dose of IR. **(F)** Normalized clonogenic survival after increasing concentration of etoposide (Etop.) alone or combined with HT and/or HSP90-*i*. Bar graphs (right-hand side panels outlined by dashed lines) show the survival at 3.3 μM concentration of Etoposide.

Since HSP90 inhibition enhances the inhibitory effects of hyperthermia on DSB repair, we investigated whether Ganetespib can potentiate cytotoxicity of treatment combining various DSB-inducing agents (radiation and chemotherapy) with hyperthermia. We focused on ionizing radiation (IR), cisplatin, gemcitabine and etoposide – chemotherapeutics that are known to induce DSBs and that are relevant in clinical cancer treatment [21–23]. We performed clonogenic survival assays to measure the effects of hyperthermia and/or Ganetespib on the cytotoxicity of these agents (Figure 3). IR directly induces DSBs that are then repaired by NHEJ or HR, depending on the cell cycle phase and hyperthermia has been shown to sensitize cancer cells and tumors to IR [24]. When combined with IR alone, Ganetespib did not induce additional cytotoxicity (Figure 3B), suggesting that short inhibition of HSP90 is insufficient for detectable downregulation of DSB repair. However, we detected a statistically significant decrease in survival after addition of Ganetespib to IR when it was combined with hyperthermia, even at the relatively low dose of 2 Gy, in both cell lines (Figure 3B). Cisplatin is an effective DNA cross-linking agent and repair of cisplatin-induced lesions in replicating cells requires HR and nucleotide excision repair [25, 26]. Hyperthermia has been reported to be a strong sensitizer to cisplatin [27], which is confirmed by our results (Figure 3C). This was in contrast to Ganetespib treatment alone, which did not enhance cytotoxicity of cisplatin, similar to that of IR. However, Ganetespib further enhanced cytotoxicity of the cisplatin and hyperthermia combination treatment, at least at cisplatin concentrations exceeding 0.9 μM (Figure 3C). Gemcitabine is a clinically-applied nucleoside analog that directly targets HR [28, 29], but its main mechanism of action involves inhibition of DNA synthesis [30], which can lead to collapse of replication forks and induction of DSBs [31]. We found that the cytotoxicity of a 24 h incubation period with gemcitabine was generally potentiated by hyperthermia, but not by Ganetespib alone (Figure 3D). A combination of hyperthermia, gemcitabine and Ganetespib, however, significantly decreased cell survival, as compared to the hyperthermia + gemcitabine combination treatment. This was observed at nearly all tested concentrations of gemcitabine, in both HeLa and SiHa cells (Figure 3D).

Since our earlier results showed that Ganetespib can shorten the time of hyperthermia treatment that is required for efficient BRCA2 degradation (Figure 1C), we speculated that HSP90 inhibition can also enhance radiosensitization by short hyperthermia treatments. Remarkably, we indeed found that a 30 min hyperthermia + Ganetespib combination sensitized HeLa and SiHa cells to a similar degree as a 60 min exposure to heat alone (Figure 3E).

Finally, we tested whether Ganetespib potentiates cytotoxicity of etoposide, an inhibitor of topoisomerase 2 (TOP2), which blocks the TOP2/DNA cleavage complexes, leading to DSB formation [32]. Hyperthermia did not sensitize SiHa cells to etoposide, and there was only a moderate sensitization in HeLa cells, at concentrations exceeding 1 μM (Figure 3F). HeLa cells were similarly sensitized by addition of Ganetespib alone or hyperthermia alone. The combination of Ganetespib and hyperthermia did not decrease cell survival in SiHa cells and only slightly (and not significantly, *p* = 0.09) in HeLa cells. Combined, these results suggest that chemical inhibition of HSP90 can potentiate the cytotoxicity of combinational approaches including hyperthermia and some, but not all, chemotherapeutic agents that inflict DNA damage. At the same time, it is apparent that the treatment with Ganetespib alone, at concentrations that stimulate cytotoxic effects of hyperthermia, does not induce significant toxicity *in vitro*.

### Inhibition of HSP90 combined with hyperthermia and IR or cisplatin affects cell cycle progression and cell fate

**Figure 4:**
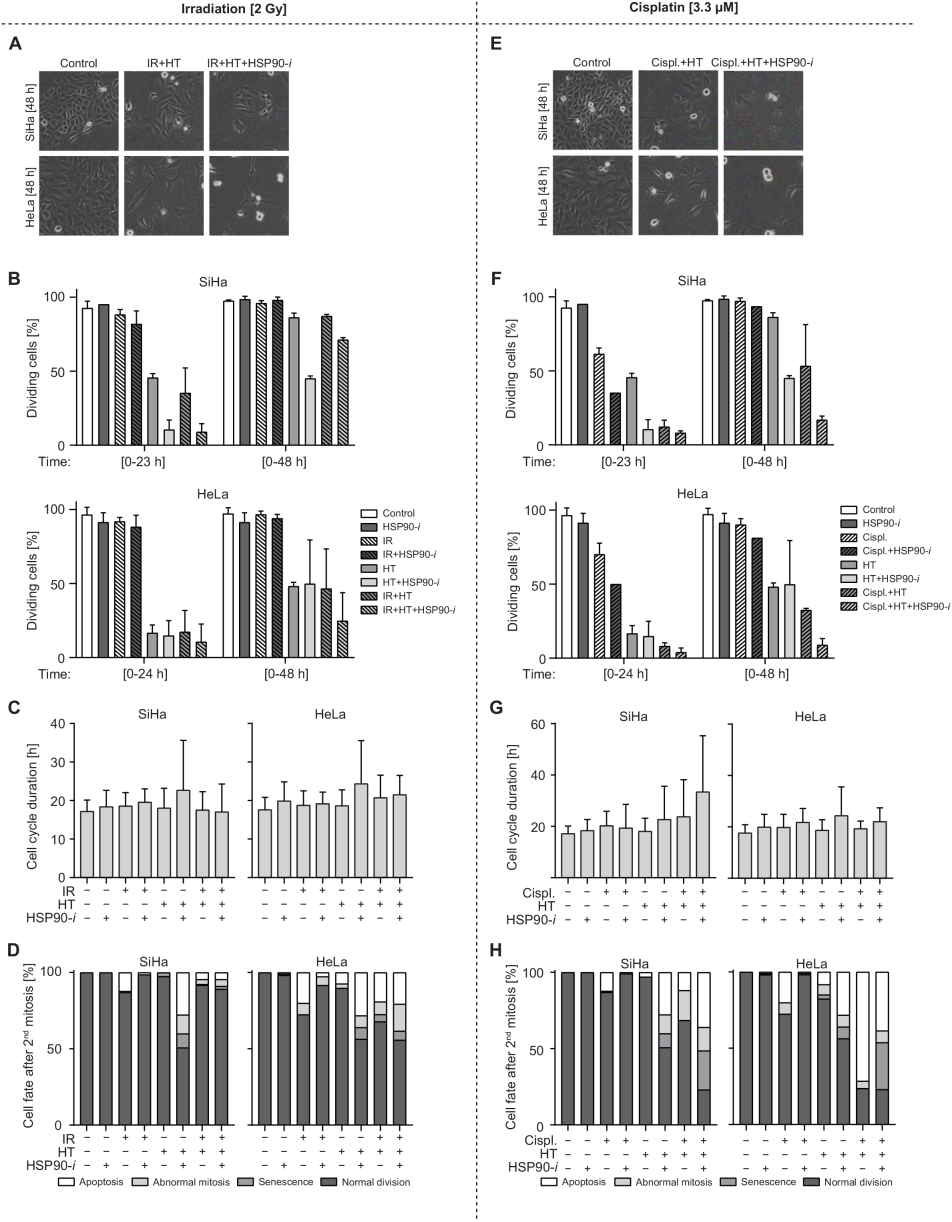
Treatments combining inhibition of HSP90 with hyperthermia (HT) and IR/cisplatin affect the cell cycle progression and cell fate **(A-D)** Cells were preincubated with vehicle or 30 nM Ganetespib (HSP90-*i*) for 30 min at 37°C, then mock-treated or exposed to 2 Gy of γ-radiation and incubated at 37 (control) or 42°C (HT) for one hour. Medium was then refreshed and cells were imaged for 96 h at intervals of 15 min. **(E-H)** Cells were preincubated with vehicle or 30 nM HSP90-*i* and/or 3.3 μM cisplatin (Cispl.) for 30 min at 37°C and then incubated at 37 (control) or 42°C (HT) for 60 min. Medium was refreshed and cells were imaged for 96 h at time intervals of 15 min. (A, E) Representative pictures of SiHa and HeLa cells at 48 h after the indicated treatments. (B, F) Average percentage of cells that successfully divided within a single (23h for SiHa and 24h for HeLa) or double (48 h) cell cycle time (+ two standard deviations). (C, G) Cell cycle times measured as the time between the first and second mitosis after the indicated treatments. (D, H) Fate of the daughter cells directly after the second mitosis. The numbers of cells analyzed in each individual treatment/measurement group are shown in Supplementary Figure 2.

To further explore how HSP90 inhibition enhances the cytotoxic effects of hyperthermia in combination with radiation and chemotherapy, we recorded time-lapse movies of HeLa and SiHa cells after single-agent and different combinational treatments. We focused on IR and cisplatin because these modalities are often combined with hyperthermia for treatment of cervical cancer [33, 34] and because our results indicated that HSP90 inhibition generally enhances the cytotoxicity of these agents when combined with hyperthermia (Figure 3). All treatment protocols mirrored those used for measuring the clonogenic survival (Figure 3A), except after refreshment of the medium cells were transferred to a live-cell microscope and time-lapse images were recorded for up to 96 h. Sample images captured after selected treatments are shown in Figure 4A and 4E. We measured various parameters related to cell cycle progression, cell division and cell fate. First, we determined the average duration of the cell cycle under normal conditions, in both cell lines (Supplementary Figure 2A). Next, we focused on the DNA-damaging agent IR and quantified the percentage of treated and control cells that were able to enter mitosis within a single or double cell cycle time (plus two standard deviations) after the treatment (Figure 4B). We observed that treatment without hyperthermia, including exposure to IR, did not significantly affect cell cycle progression, since nearly all cells were able to enter mitosis within the first 23 h after treatment. In contrast, hyperthermia-based treatments reduced the percentage of cells that entered mitosis during the first 23 or 48 h. The largest reduction was observed after the hyperthermia + Ganetespib combination, with or without IR. Additionally, hyperthermia-based treatments increased the frequency of abnormal first mitoses (Supplementary Figure 2B). These differences were not accompanied by an altered duration of the cell cycle in cells that successfully completed the first mitosis (Figure 4C) but the frequencies of these cells were strongly reduced after hyperthermia-based double and triple-combinational treatments (Supplementary Figure 2D). Furthermore, these treatments generally caused considerably increased frequencies of abnormal cell division, senescence and apoptosis (Figure 4D). One notable exception was the triple-modality treatment of SiHa cells, which did induce substantial cell cycle delay (Figure 4B) but did not cause abnormalities in those cells that were able to successfully divide (Figure 4D).

Treatments with cisplatin showed even stronger effects of double and triple modalities involving hyperthermia and, interestingly, of the cisplatin + Ganetespib double treatment. This was apparent in quantification of successful cell divisions (Figure 4F), abnormal first mitosis (Supplementary Figure 2C) and of cell fate after the second mitosis (Figure 4H). Also here, frequencies of these cells were strongly reduced after hyperthermia-based double and triple-combinational treatments (Supplementary Figure 2E). Similarly to experiments involving IR, most combinations, except for triple modality in SiHa cells, did not considerably affect the length of the first cell cycle after treatment (in those cells that were able to successfully divide) (Figure 4G). Importantly, the triple combination regimen was clearly superior in causing disturbance of the cell cycle and mitosis as well as apoptosis and senescence. In conclusion, these observations generally confirm that HSP90 inhibition potentiates cytotoxicity of combinational treatments including hyperthermia and cisplatin/IR. They also provide further details on how this toxicity is manifested in living cells.

### Inhibition of HSP90 reduces thermotolerance

**Figure 5:**
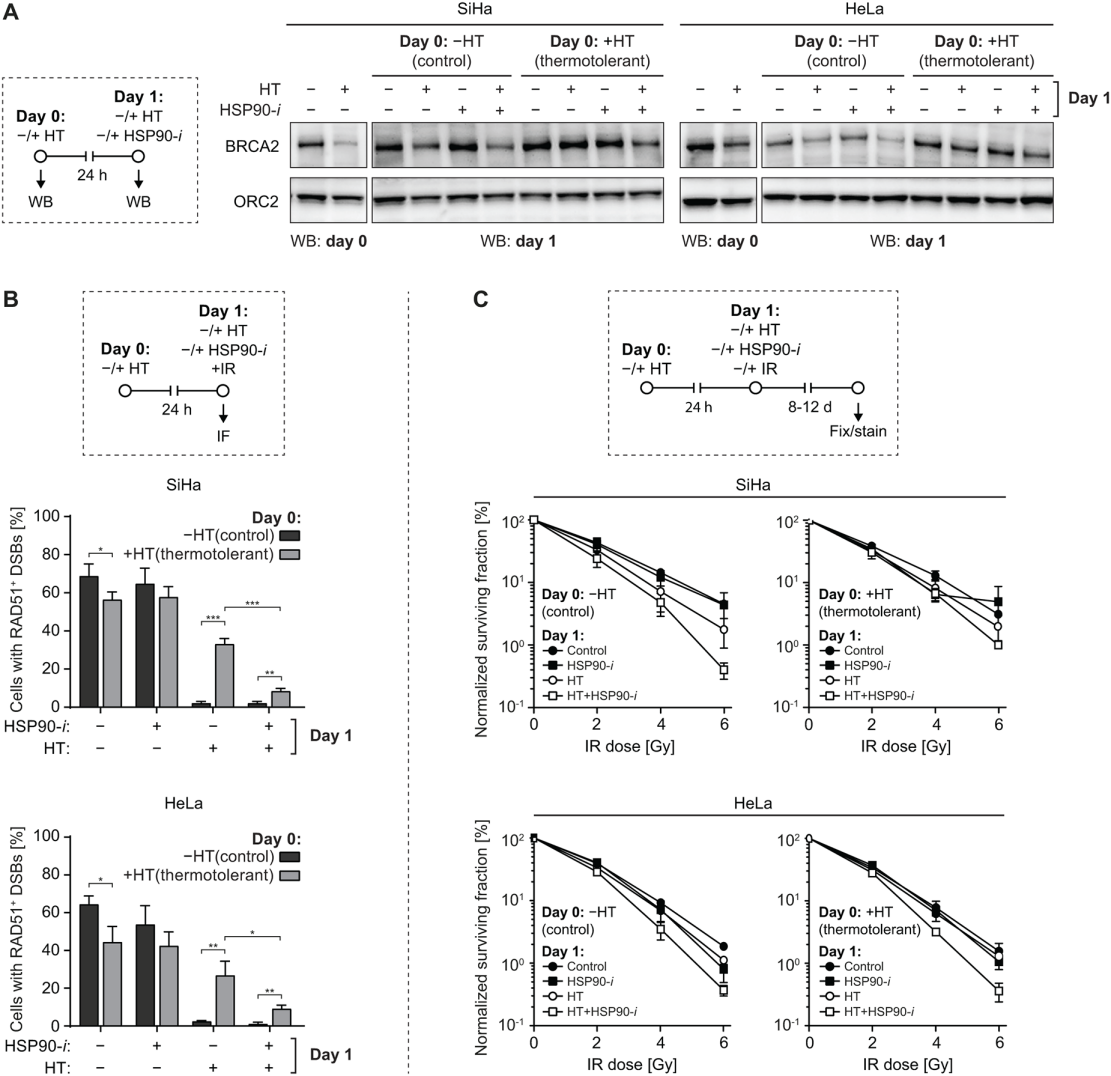
Inhibition of HSP90 reduces thermotolerance **(A)** SiHa or HeLa cells were sham-treated or rendered thermotolerant by incubation for 1 h at 37 or 42°C (HT), respectively. Some of the treated cell samples were immediately lysed and analyzed by Western blotting to confirm the effectiveness of hyperthermia (HT) treatment by analyzing BRCA2 levels (panels marked by ‘WB: day 0’). One day later, the remaining samples were subjected to a second HT treatment, in the absence or presence of 30 nM Ganetespib (HSP90-*i*), lysed, and BRCA2 levels were determined by Western blotting (panels marked by ‘WB: day 1’). The experimental design is schematically represented in the left-hand panel. ORC2 was used as a loading control. **(B)** SiHa or HeLa cells were treated as described in (A), except immediately after the second HT treatment cells were irradiated using α-particles, fixed 30 min later and immunostained against γH2AX and RAD51. Graphs represent the average percentage of cells containing α-particle induced tracks of γH2AX foci that were also positive for RAD51. The experimental design is schematically represented in the top panel. **(C)** Cells were rendered thermotolerant as in (A). 24 h later the cells were trypsinized, counted and seeded into 6-well plates. 4 h after seeding, they were incubated for 1 h at 37 or 42°C, in the absence or presence of HSP90-*i,* and exposed to the indicated dose of IR. Normalized clonogenic survival was determined 8 (HeLa) or 12 (SiHa) days later. The experimental design is schematically represented in the top panel.

Thermotolerance is a hyperthermia-induced state of resistance to subsequent hyperthermia treatments, driven – at least partially – by expression of HSPs, including HSP70 and HSP90 [35]. It has been shown earlier that benzoquinone ansamycin inhibitors of Hsp90 can delay the recovery from heat stress and suppress some aspects of thermotolerance in *Drosophila* [36]. To establish whether Ganetespib can affect these processes in mammalian cells, we first evaluated the effects of thermotolerance on hyperthermia-mediated reduction of BRCA2 levels. To induce thermotolerance, we first treated SiHa and HeLa cells for 1 h at 37 or 42°C. 24 h later, we exposed these cells to a second hyperthermia treatment, with or without Ganetespib, and analyzed levels of BRCA2 by Western blotting (Figure 5A). As expected, in control (non-thermotolerant) cells, hyperthermia reduced BRCA2 levels and Ganetespib exacerbated this effect, while hyperthermia failed to induce BRCA2 degradation in cells pre-treated at 42°C, confirming their state of thermotolerance. However, addition of a HSP90 inhibitor partially abolished this effect, as BRCA2 levels in thermotolerant cells heated in the presence of Ganetespib were reduced, albeit not as dramatically as in non-thermotolerant cells (Figure 5A).

Thermotolerance was also manifested by a significantly reduced ability of hyperthermia to inhibit RAD51 foci formation in cells heated 24 h before alpha-particle irradiation (Figure 5B, compare bars 5 and 6 in each panel). However, even in thermotolerant cells, RAD51 focus formation was inhibited nearly as effectively as in non-thermotolerant cells when they were heated in the presence of Ganetespib (Figure 5B, compare bars 6 and 8).

Finally, we investigated whether Ganetespib can reduce clinically-relevant effects of thermotolerance. We observed that hyperthermia-mediated radiosensitization of thermotolerant cells was reduced, as compared to their non-thermotolerant counterparts, but it could be restored by the addition of Ganetespib (Figure 5C). In summary, our results demonstrate that HSP90 inhibition can reduce multiple aspects of thermotolerance in cervix cancer cell lines.

## DISCUSSION

Strategies for efficient and targeted inhibition of DNA repair can help to improve clinical cancer therapies that rely on induction of DNA damage to destroy malignant cells. One example of such strategy is inhibition of PARP1, a protein involved in repair of SSBs, DSBs and in the regulation of the chromatin environment [37]. PARP1 inhibitors are used to target HR-deficient tumors, while sparing HR-proficient healthy tissues, in a reframed synthetic-lethality approach [38, 39]. Our previous *in vitro* and *in vivo* results suggested that mild hyperthermia in clinically-obtainable temperature range (41-42.5°C) can be used for on-demand induction of HR deficiency in cells and tissues [7] and implied that HSP90 inhibition can potentiate this effect. In the current study, we further explored this hypothesis and show that Ganetespib – a new-generation HSP90 inhibitor – enhances hyperthermia-mediated degradation of BRCA2 and inhibition of HR (Figure 1). These data are in line with studies showing that BRCA2 is a client of HSP90 and that HSP90 inhibition affects BRCA2 stability [40]. In addition to BRCA2, HSP90 manages multiple other proteins associated with DNA repair and the stability of these factors is affected by inhibition of HSP90 [13, 40, 41]. This is especially interesting because we have shown earlier that DSB repair is shunted to NHEJ upon HR inhibition by HT [42] and that protein levels of the major NHEJ players DNA-PK and LIGIV increase after HT treatment [10, 43]. Since HSP90 inhibition decreases DSB-induced phosphorylation of DNA-PK and induces degradation of the alternative-NHEJ factor XRCC1 [13], HSP90 inhibition could limit the functionality of NHEJ after HT, further increasing DSB cytotoxicity. The combination of a HSP90 inhibitor with hyperthermia thus emerges as a self-reinforcing strategy to disable DSB repair, creating on-demand conditions of *stimulated synthetic lethality*.

Except for the effects on HR, our results suggest induction of DSBs after longer periods of time (16-48 h) after hyperthermia and – to a larger degree – after hyperthermia + Ganetespib treatment (Figure 2). In particular, the highly increased MN formation is a strong indicator of cells entering mitosis with unrepaired DSBs [20]. Induction of MN formation by hyperthermia has been reported over three decades ago [44] and confirmed more recently [45], but its stimulation by HSP90 inhibition is a novel observation. The late appearance of MN and γH2AX foci suggests that they are not directly induced by treatment, but rather arise with the progression of the cell cycle. This is in line with the previously advanced hypothesis that the induction of DSBs after hyperthermia is caused by inhibition of TOP1, leading to formation of SSBs [18]. Unrepaired SSBs can then derail replication forks and result in DSB formation in the next S-phase. Such one-ended DSBs are likely similar to those hypothetically induced after PARP1 inhibition. Since collapsed replication forks require HR for repair, inhibition of HR by hyperthermia contributes to the resulting toxicity, but this contribution may be limited by the temporary and reversible character of hyperthermia-mediated HR suppression (Figure 1B).

The observation of enhanced and prolonged HR inhibition by hyperthermia + Ganetespib invites the combination of *stimulated synthetic lethality* with induction of DSBs in the temporary therapeutic window of HR deficiency. This is clearly supported by our results showing potentiation of hyperthermia-induced radiosensitization and chemosensitization by Ganetespib (Figures 3 and 4). Similar to late DSB induction by hyperthermia + Ganetespib treatment, clonogenic cell death and late effects on the cell cycle and division capabilities are observed when treatment is combined with DSB-inducing agents including IR, cisplatin and gemcitabine. In contrast to these agents, we do not detect significant thermal sensitization of cells to the TOP2 inhibitor etoposide, whether or not Ganetespib is present during hyperthermia treatment. This can be explained by the previously described inhibitory effects of hyperthermia on the formation of TOP2 cleavage complex, which may reduce the efficiency of DSB induction and treatment cytotoxicity [46].

**Figure 6:**
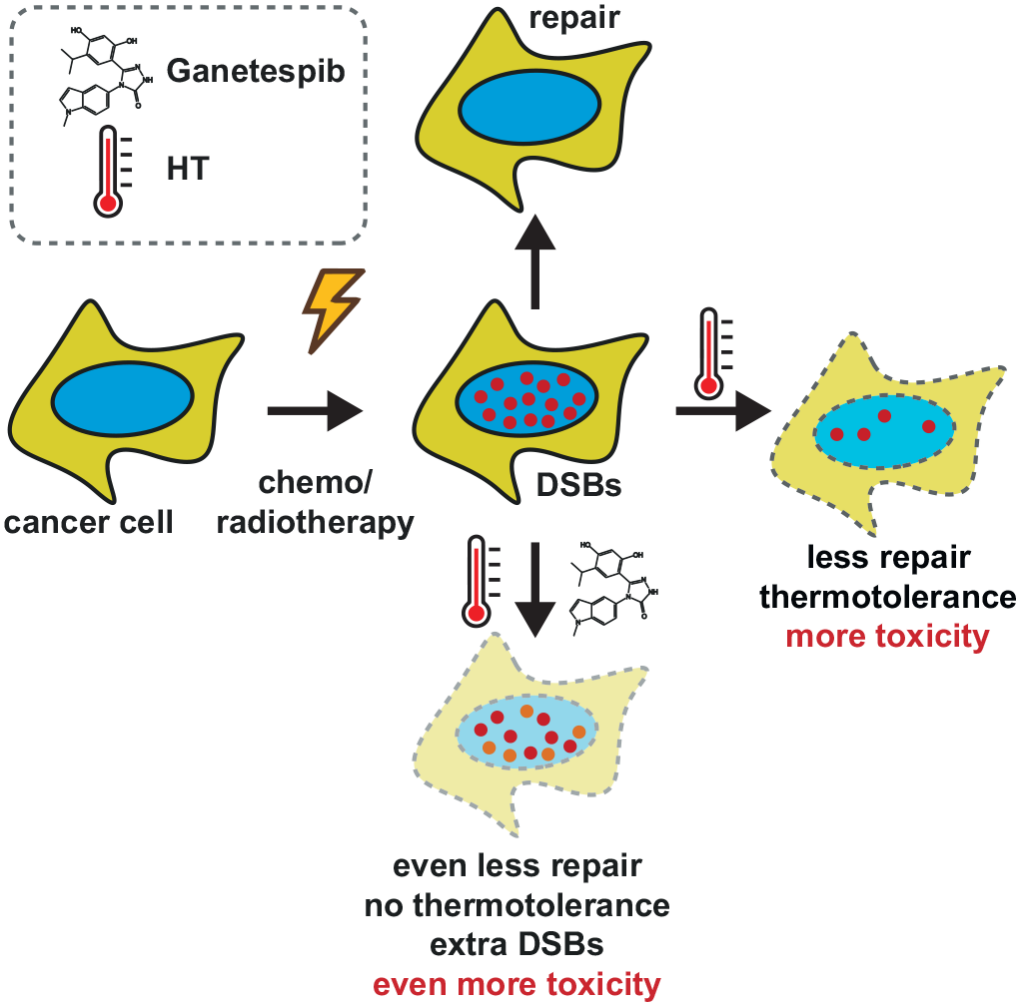
Schematic representation of combination therapies involving DNA-damaging agents, HT and HSP90 inhibitor

Our results show at least three different aspects of treatments comprising HSP90 inhibition and hyperthermia that can be beneficial in cancer treatment (Figure 6). First, the treatments produce DSBs in dividing cells and likely also cause cytotoxicity by other mechanisms. In nearly all experiments, we observed that Ganetespib considerably potentiates cell killing by hyperthermia, in line with a recent study that reported enhancement of the effects of hyperthermia by 17-DMAG [47, 48]. It is worth noting that prolonged administration of Ganetespib alone can also sensitize cancer cells to radiation and some chemotherapeutic drugs [49–51]. Second, the increased inhibition of HR (and, potentially, other DNA repair mechanisms) by hyperthermia and Ganetespib sensitizes cells to multiple DSB-inducing agents, indirectly increasing their cytotoxicity. Third, Ganetespib could be potentially used to reduce thermotolerance and thus enable more frequent hyperthermia treatments. The relative importance of the first two effects for the final cell killing is currently unclear but DSB induction may contribute to therapy efficacy in tumor areas where chemotherapeutics or radiation do not reach all cancer cells, while sensitizing effects could dominate in cells exposed to sufficiently high doses of DNA damaging agents.

Importantly for clinical application, our results imply that a single, short pulse of Ganetespib, combined with hyperthermia, is sufficient for a temporary but considerable downregulation of HR (Figure 1) [7]. This approach is in conceptual opposition to the long-term application of Ganetespib that has been tested in clinical trials [14], including the phase III trial of Ganetespib in combination with docetaxel which failed in patients with advanced non-small cell lung cancer (https://clinicaltrials.gov/ct2/show/NCT01798485). Long-term exposure to Ganetespib has been found to be well tolerated [14] and the single application that is required for boosting hyperthermia efficacy should be safe in clinical practice. Strategies allowing potentiation of the cytotoxic and sensitizing effects of hyperthermia can lead to improved therapy outcomes via multiple avenues, e.g. by inducing stronger cytotoxicity while sparing the non-heated healthy tissues, by allowing reduction of the required dose of DNA-damaging agents or by rendering hyperthermia treatments effective at decreased temperatures or shorter durations. Our study suggests that inhibition of HSP90 is one such strategy, with limited systemic side-effects, paving the way for rational design of improved hyperthermia treatment protocols and for *in vivo* studies involving animal models.

## MATERIALS AND METHODS

### Cell lines and cell culture

SiHa and HeLa cervical cancer cell lines were obtained from the American Type Culture Collection (ATCC) and cultured in EMEM medium (Lonza) enriched with 10% fetal bovine serum (FBS) and 1% of penicillin/streptomycin (Gibco, 10000 U/mL). Cells were maintained at 37°C, in an atmosphere containing 5% CO_2_. During the experiments with hyperthermia, cells were incubated for 65 min at 42°C in an atmosphere containing 5% CO_2_ (the medium in the wells needed approx. 5 min to reach the target temperature of 42°C). For experiments involving α-particles, cells were cultured in custom-made dishes with 4 μm-thick polypropylene bottom, as described earlier [52].

### Chemical agents, hyperthermia treatments and irradiation

Cells were treated with the indicated concentrations of Ganetespib (STA-9090, Synta Pharmaceuticals), cisplatin (cDDP; Platosin®, Pharmachemie), gemcitabine (Actavis) and etoposide (Sigma Aldrich). Ganetespib and cisplatin were added 30 min before and removed immediately after the end of hyperthermia treatments, by washing cells and adding fresh medium. Gemcitabine was added 24 h before the start of hyperthermia experiments. Hyperthermia was applied by partially submerging cell culture dishes in a calibrated water bath, at the appropriate CO_2_ concentration. The temperature was monitored by a thermocouple directly in cell culture dishes. To allow for temperature increase from 37 to 42°C, cells were always incubated 5 min longer than what is indicated in the text/figures. In experiments involving irradiation, cells were exposed to the indicated doses from a ^137^Cs γ-ray source (∼0.5 Gy/min) or from an ^241^Am α-particle source. To produce linear tracks of DSBs, cells were irradiated through the polypropylene bottom of culture dishes for 1 min, with the α-particle source positioned under an angle of approximately 45° below the bottom of the dish, as described previously [52].

### Immunohistochemistry

At the indicated time points after irradiation, cells were fixed with 2% paraformaldehyde in PBS for 15 min at room temperature. Fixed samples were washed twice with PBS and incubated in TNBS (PBS supplemented with 1% FCS and 0.1% Triton-X100) for 30 min. Samples were then incubated with the primary mouse anti-γH2AX (1:100, Millipore) and rat anti-Rad51 (1:50) antibodies diluted in TNBS for 2 h*.* After being washed twice in TNBS, samples were incubated with the secondary anti-mouse-Cy3 and anti-rat-FITC (both 1:100, Jackson ImmunoResearch Laboratories) diluted in TNBS, for 1 h. Finally, mounting gel containing DAPI (Thermo Scientific) was added and samples were covered with glass coverslips. Slides were imaged and scored using the wide-field fluorescence microscope (DM-RA and DM-RXA, Leica).

### Clonogenic assays

24 h before treatment, 2x10^6^ cells were seeded into a 10 cm dish. On the day of the experiment, cells were trypsinized, counted and plated in triplicates of two densities per condition in 6-well plates. After 4 to 6 h incubation required for cell attachment, cells were treated with 30 nM Ganetespib for 90 min with or without cisplatin or etoposide. After the first 30 min of incubation at 37°C, plates were either transferred to a 42°C water bath or were incubated at 37°C for the remaining 65 min. In clonogenic survival experiments involving ionizing radiation (IR), cells were irradiated after the 30 min treatment with Ganetespib, immediately prior to the hyperthermia treatment. Directly after hyperthermia, cells were washed with PBS and incubated in fresh medium for 8 (HeLa) or 13 (SiHa) days. Next, colonies were fixed, stained and counted. In experiments involving gemcitabine, cells were treated for 24 h, starting directly after plating into 10 cm dishes and until the start of the experiment (0 h). A schematic overview of the treatment schedules is depicted in Figure 3A.

### Time-lapse microscopy

24 h prior to the indicated treatments, cells were plated in 6- or 12-well plates at a density of 15,000 or 7,000 cells per well, respectively. After treatment, cells were washed with PBS and fresh medium was added. The medium was covered with a layer of mineral oil (Sigma-Aldrich) to prevent evaporation during imaging. Cells were imaged for 96 h, at intervals of 15 min, using a wide-field phase-contrast microscope (Leica). The cell cycle time was defined as the time between the first and second successful mitosis observed after treatment; senescence was absence of cell division for at least 48 h and abnormal mitosis was division that gave rise to abnormal progeny.

### Western blotting

Cells were harvested immediately after treatments and lysed in Laemmli sample buffer (4% SDS, 20% glycerol and 120 mM Tris pH 6.8). Protein levels were quantified with the Lowry protein assay. A total of 50 μg protein supplemented with bromophenol blue and β-mercaptoethanol was loaded and separated on a NuPage 3-8% Tris-Acetate protein gel (Thermo Fisher Scientific). The protein samples were transferred to a PVDF membrane, incubated for 1 h in blocking buffer at 4°C (PBS with 0.05% Tween (PBS-T) and 3% nonfat dry milk) and then overnight with the primary mouse anti-BRCA2 (OP95-Ab-1, Merck Millipore), rabbit anti-cyclin A (C-19, Santa Cruz Biotechnology) or mouse anti-ORC2 (ab68348, Abcam) antibodies (diluted 1:1000 in blocking buffer). The membrane was washed five times for 8 min with PBS-T and incubated with the relevant secondary antibodies (horseradish peroxidase (HRP)-conjugated sheep anti-mouse or donkey-anti rabbit IgG, both 1:2000 in blocking buffer, Jackson ImmunoResearch Laboratories) for 2 h at room temperature. The proteins on the membrane were visualized with enhanced chemiluminescence (ECL) substrate and imaged with the Alliance imager 4.7 (Uvitec).

### Data collection and statistics

The unpaired *t*-test was used for inter-group comparisons of the means. Graphs presented in Figure 4 show results of two independent experiments that each included both IR (B-D) and cisplatin (G-I) arms. Therefore, the same results obtained from control as well as single and double-agent treatments involving HT and HSP90-*i* are shown in both arms. In the case of the cisplatin+HSP90-*i* treatment, n was 1. The numbers of cells analyzed in these experiments for each panel of Figure 4 (1340 HeLa cells and 1850 SiHa cells in total) are presented in Supplementary Figure 2D and 2E. In Figure 3C, when 33 μM cisplatin was combined with hyperthermia and HSP90-*i,* n = 1 because no colonies could be detected at this concentration in the remaining experiments. All other graphs summarize the results of at least three independent experiments, with error bars indicating standard deviation. Asterisks indicate statistical significance with the *p*-values as follows: ^*^ < 0.05, ^**^ < 0.01, ^***^ < 0.001.

## SUPPLEMENTARY MATERIALS FIGURES


