# Supplementary material for: Boosting the effects of hyperthermia-based anticancer treatments by HSP90 inhibition
Source: Oncotarget. 2017 Oct 27;8(57):97490–503. doi: 10.18632/oncotarget.22142 (PMC5722578; doi:10.18632/oncotarget.22142)
Supplement: Supplementary file 1 [file oncotarget-08-97490-s001.pdf]

Boosting the effects of hyperthermia-based anticancer treatments by HSP90 inhibition

SUPPLEMENTARY MATERIALS

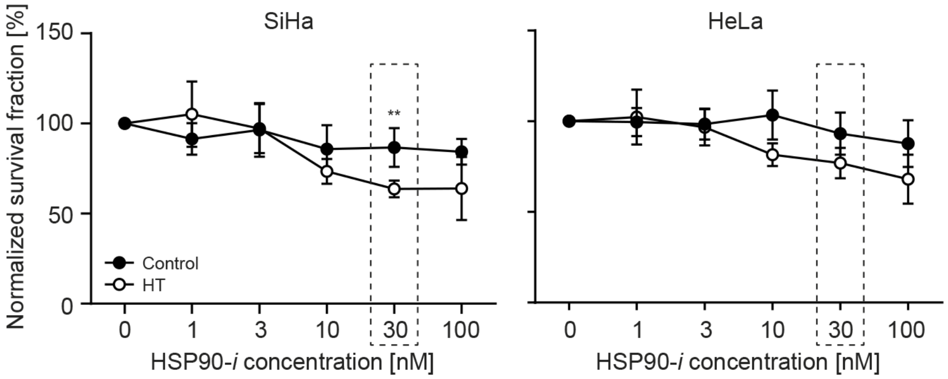

Supplementary Figure 1: HSP90 inhibitor Ganetespib potentiates the cytotoxic effects of hyperthermia (HT). Normalized clonogenic survival fraction after a 1.5 h incubation at increasing concentrations of Ganetespib (HSP90-*i*) at 37 (control) or 42°C (HT). 30 nM (marked by the dashed box) was selected as working concentration for the remaining experiments.

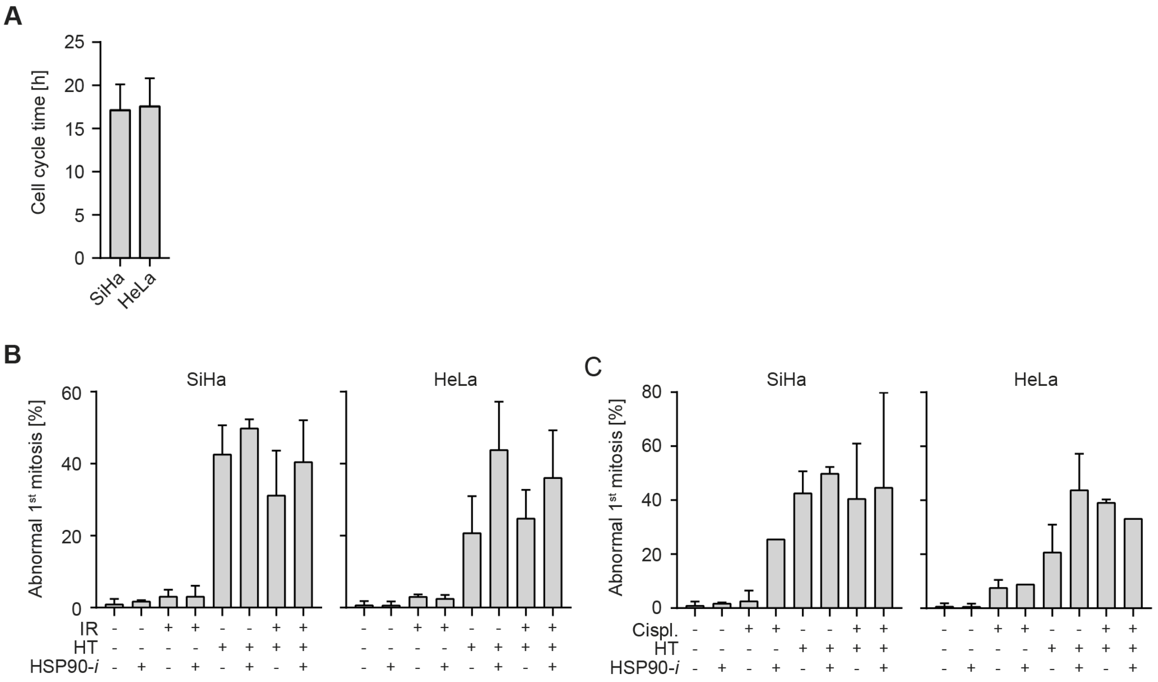

D

Irradiation [n]	Figure 4B SiHa	Figure 4B HeLa	Figure 4C SiHa	Figure 4C HeLa	Figure 4D SiHa	Figure 4D HeLa
Control	248	141	255	211	255	211
HSP90-*i*	226	158	127	113	127	114
IR	252	233	146	153	148	157
IR+HSP90-*i*	296	212	156	165	160	178
HT	167	98	101	32	115	40
HT+HSP90-*i*	133	93	41	25	65	39
IR+HT	262	170	150	64	158	84
IR+HT+HSP90-*i*	266	165	147	25	157	34

E

Cisplatin [n]	Figure 4F SiHa	Figure 4F HeLa	Figure 4G SiHa	Figure 4G HeLa	Figure 4H SiHa	Figure 4H HeLa
Control	248	141	255	211	255	211
HSP90-*i*	226	158	127	113	127	114
Cispl.	259	128	127	120	128	121
Cispl.+HSP90-*i*	125	96	66	66	68	74
HT	167	98	101	32	115	40
HT+HSP90-*i*	133	93	41	25	65	39
Cispl.+HT	171	86	90	6	102	21
Cispl.+HT+HSP90-*i*	189	100	15	4	39	13

Supplementary Figure 2: Treatments combining inhibition of HSP90 with hyperthermia (HT) and IR/cisplatin affect cell cycle progression and cell fate. Cells were treated and imaged as described in legend of Figure 4. **(A)** Average cell cycle time of untreated SiHa and HeLa cells. **(B, C)** Average percentages of cells that experienced abnormalities during the first mitosis following the treatment. **(D, E)** Tables showing the numbers of cells analysed in each individual treatment group (data from these analyses is presented in Figure 4). Notably, the numbers of cells that could be adequately analyzed are dramatically reduced in the double/triple treatment groups.
